# Novel Use of Household Items in Open and Robotic Surgical Skills Resident Education

**DOI:** 10.1155/2019/5794957

**Published:** 2019-03-07

**Authors:** Keri Rowley, Deepak Pruthi, Osamah Al-Bayati, Joseph Basler, Michael A. Liss

**Affiliations:** ^1^University of Texas Health San Antonio, Department of Urology, San Antonio, USA; ^2^University of Texas Health San Antonio Cancer Center, San Antonio, USA; ^3^South Texas Veterans Healthcare System, Department of Surgery, Division of Urology, San Antonio, USA; ^4^University of Texas, College of Pharmacy, Austin, USA

## Abstract

**Background:**

The aim of this study was to investigate the effectiveness of surgical simulators created using household items and to determine their potential role in surgical skills training.

**Methods:**

Ten urology residents attended a surgical skills workshop and practiced using surgical simulators and models. These included a wound closure model, an open prostatectomy model, a delicate tissue simulation, a knot-tying station, and a laparoscopic simulator. After the workshop, the residents completed a 5-point Likert questionnaire. Primary outcome was face validity of the models. Secondary outcomes included usefulness as a training tool and ability to replicate the models.

**Results:**

All models were easily created and successfully represented the surgical task being simulated. Residents evaluated the activities as being useful for training purposes overall. They also felt confident that they could recreate the simulators.

**Conclusion:**

Low-fidelity training models can be used to improve surgical skills at a reasonable cost. The models will require further evaluation to determine construct validity and to determine how the improvements translate to OR performance. While high-fidelity simulators may continue to be utilized in formal surgical training, residents should be encouraged to supplement their training with innovative homemade models.

## 1. Background

Today's surgical trainees are increasingly challenged to improve their technical skills in an environment with reduced work hours [1]. In a field where hands-on experience is paramount to developing competency, the solution has been the utilization of surgical simulators. Many models are available for endourology as well as laparoscopic and robot-assisted urology; however, few simulators exist for open urological surgery [2, 3]. This creates additional challenges for surgical trainees, who are exposed to fewer open surgery cases due to increasing use of minimally invasive surgery techniques [4].

The current options for high-fidelity open urological surgery simulation are costly, and those that are affordable are limited to bench models for simple and suprapubic catheterization, adult male circumcision, and vasectomy [5]. Some programs have pioneered cadaveric training programs for various operations such as circumcision, vasectomy, and hydrocele repair while others have proposed improving open surgical experience by incorporating organ procurement and transplantation into the curriculum [6, 7]. While such methods are beneficial, they do not allow for repeated independent practice.

Inexpensive open urological surgery models may provide residents further opportunities to improve their open surgical skills. Urology requires mastery of both open and robotic surgical skills. Therefore, we sought to design simulators that could be easily replicated by surgical trainees at a low cost and evaluated the validity of the models for training purposes in open and robotic simulation.

## 2. Materials and Methods

### 2.1. Study Design and Data Collection

The simulators and models were used to teach surgical techniques to urology trainees at the Audie L. Murphy Memorial VA Hospital as part of a skills workshop. After the workshop, the trainees completed a 5-point Likert questionnaire to assess the models on face validity, usefulness as a training tool, and perceived ability to replicate the models (Table 1).

### 2.2. Simulators

#### 2.2.1. Wound Closure Model

**Figure 1:**
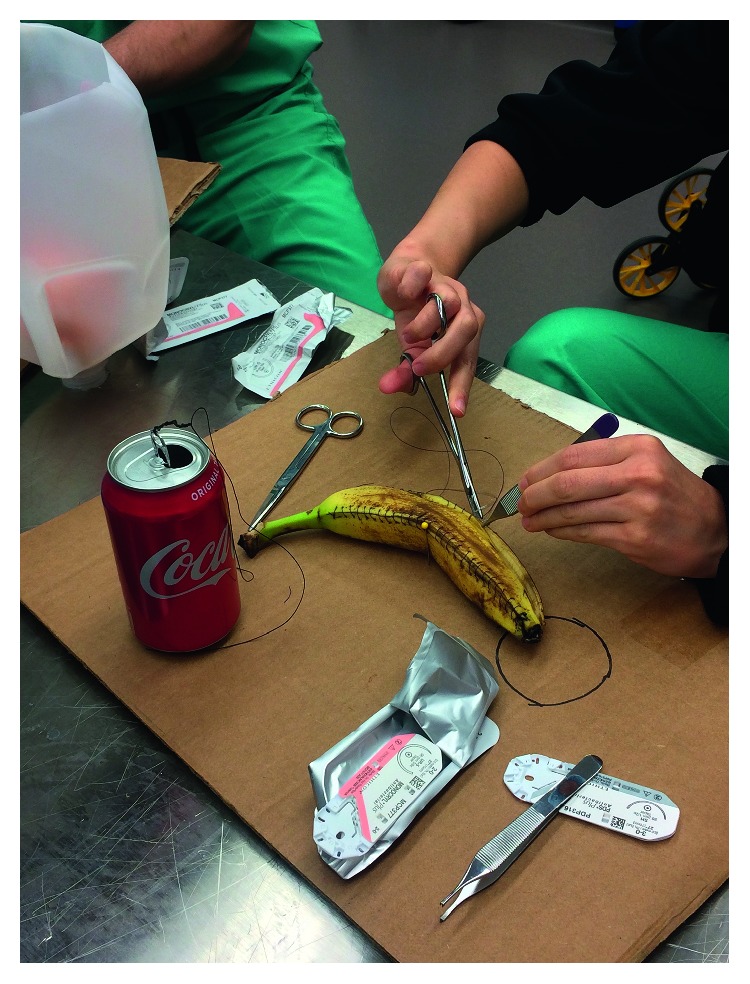
Wound closure model. Learners utilize old banana peels to practice surgical wound closure. Standard running suture and running horizontal mattress. Cola can top can be used to practice knot tying with added difficulty by reducing fluid in the can and not allowing the learner to lift the can as they lead down the knot.

The model for wound closure was created using cardboard, T-pins, and a ripe banana. The fruit was removed from the peel and secured to the cardboard surface using T-pins. Trainees practiced suturing the banana using various techniques and angles of approach (Figure 1).

#### 2.2.2. Open Prostatectomy Model

**Figure 2:**
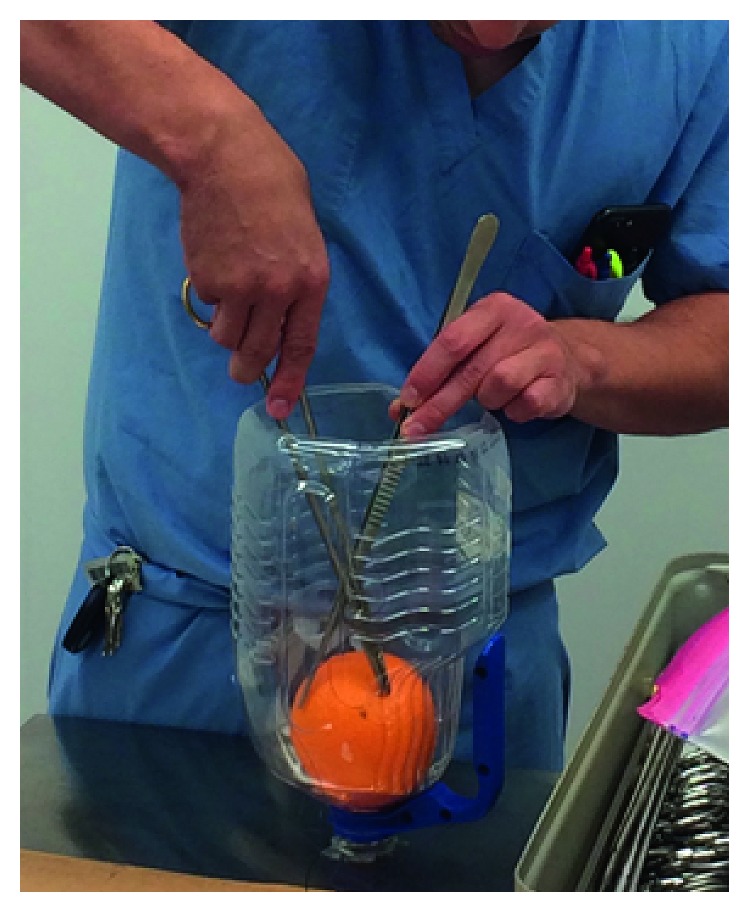
Open prostatectomy model. The orange represents the prostate, and the bottle represents a deep pelvis. Learners can use this model to practice suturing and tying in the pelvis. If the orange is glued down, the learner could also perform a simple prostatectomy by cutting the peel and removing the flesh within the orange as the transitional zone.

The model was created using a plastic 1-gallon milk or juice container and a large orange. The plastic container simulates the surgical space and abdominal cavity, and the orange represents the prostate. The bottom of the plastic container was cut out, and then, the container was inverted. Using a hot glue gun, the orange was glued into the neck of the inverted container, and the container was glued to a flat surface. The trainees were instructed to practice suturing across the surface of the orange in a straight line using DeBakey forceps and DeBakey needle holder (Figure 2). Residents also practiced knot tying in this simulated confined surgical space.

#### 2.2.3. Delicate Tissue Simulation

**Figure 3:**
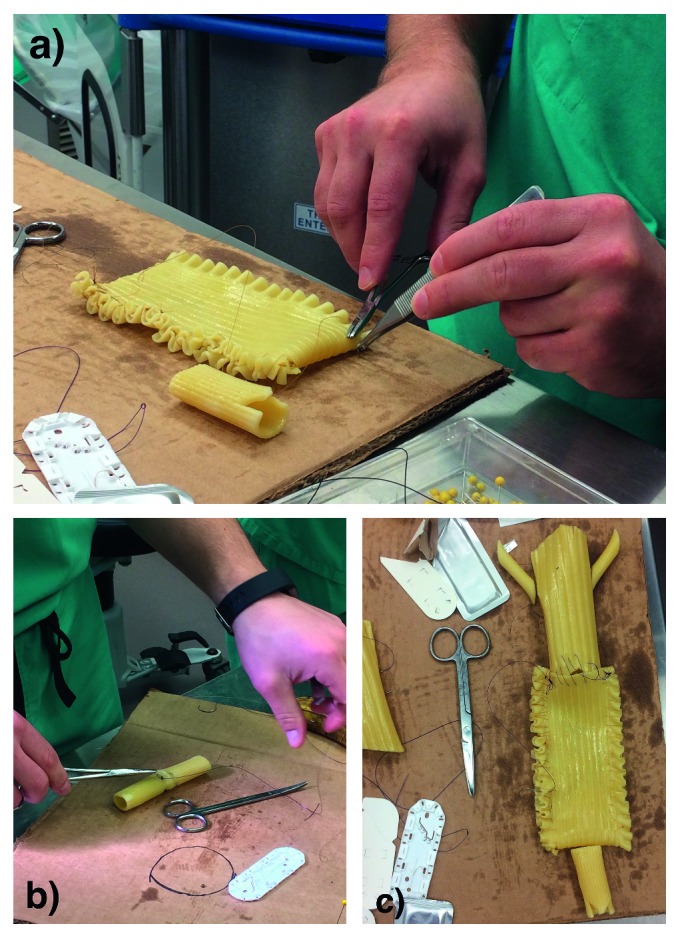
Delicate tissue simulation. (a) Resident suturing cooked pasta al dente. (b) Practicing anastomoses. (c) Neobladder recreation.

Delicate tissue was simulated using various types of boiled pasta coated in olive oil. The pasta was cooked for varying lengths of time to simulate the variation in tissue consistency. Trainees were instructed to anastomose the pasta using sutures strong enough to hold but not tear through the pasta (Figure 3). They were also advised to consider the force applied to their forceps when gripping the pasta. Cooked pasta has been previously utilized to simulate microsurgery for plastic surgery trainees [8].

#### 2.2.4. Knot-Tying Station

**Figure 4:**
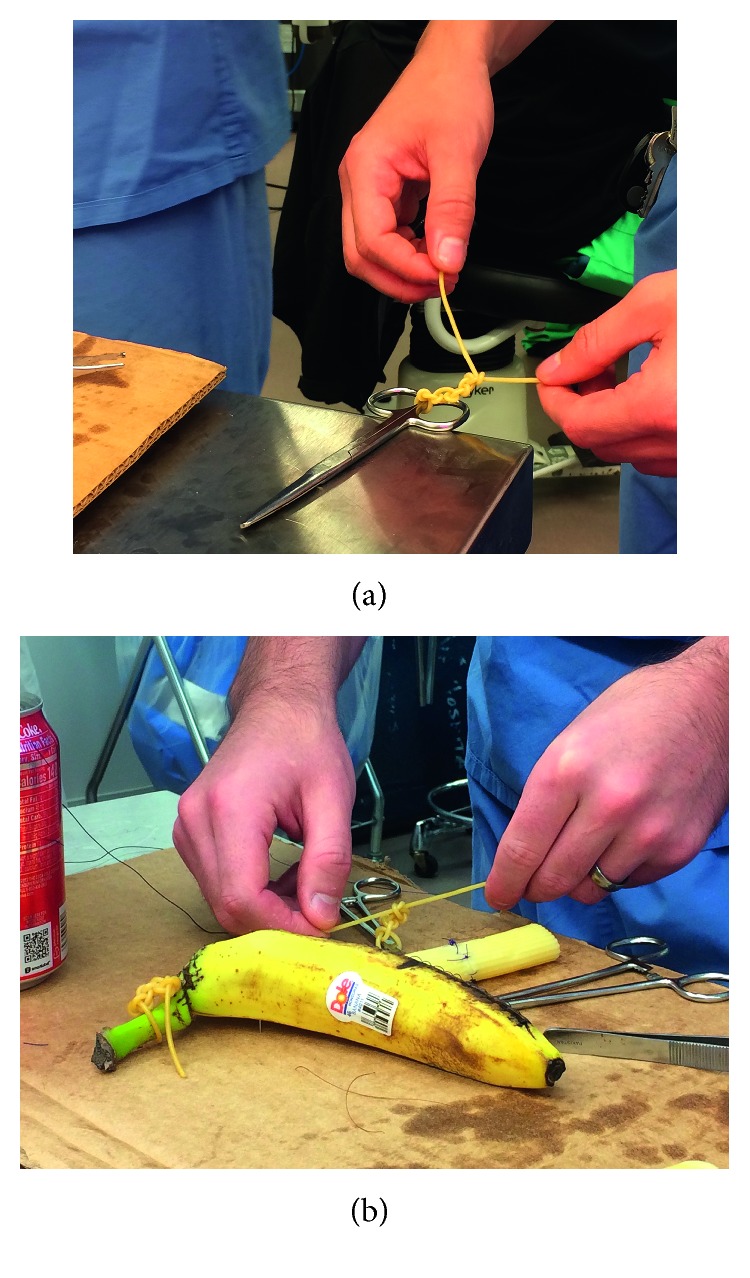
Knot tying. Utilizing overcooked and al dente spaghetti pasta to perform knot tying will allow the learner to tie a knot with appropriate pressure and confirm square knots.

This exercise utilizes cooked spaghetti noodles coated in olive oil to help trainees determine the amount of force needed to advance a knot. Trainees used one strand of noodle to tie surgical knots around various objects (Figure 4). If the noodle broke before they were able to throw a significant number of knots, they restarted with a new strand.

#### 2.2.5. Robotic Simulator

**Figure 5:**
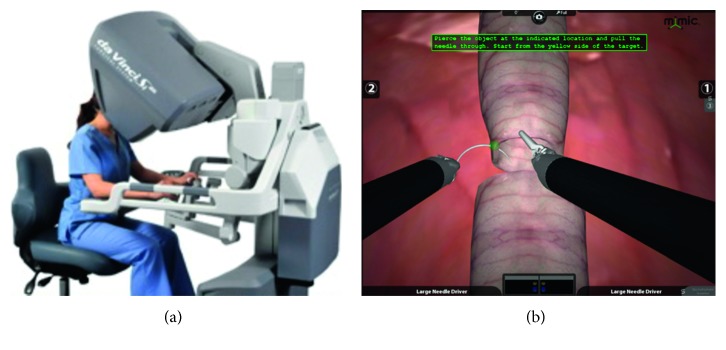
Robotic simulator by mimic technologies. The robotic simulator attaches to the back of the robot operative cart and connects to the video cable. We use the simulator function “tubes” for two rounds prior to moving onto the hands-on simulator.

**Figure 6:**
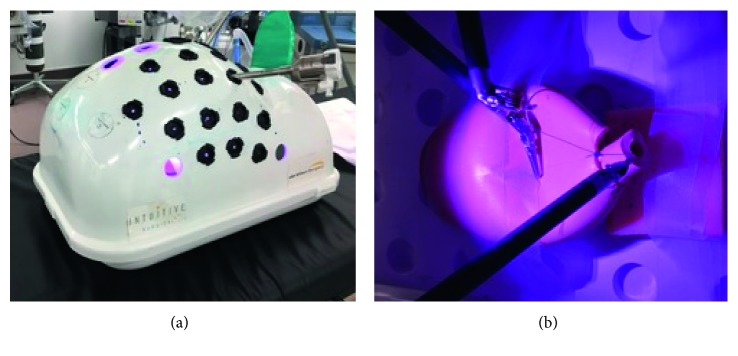
Robotic simulation (hands-on). Using the standard pelvic Da Vinci trainer and the Da Vinci Xi robot, we perform standard vesicourethral anastomosis using the LapED 4 : 1 silicone training model.

The dual console Da Vinci Xi (Intuitive Surgical, Sunnyvale, CA) was used for surgical training. One console was used for practice on the Da Vinci Surgical Skills Simulator (DVSSS) and was included to contrast the expensive high-fidelity models (Figure 5). The DVSSS uses the Mimic software program to evaluate skill competency through various exercises. Residents performed the “Thread the Rings” exercise, which primarily focuses on improving needle control. To practice robotic anastomosis, we also had the residents attempt the “tubes” function, an exercise that has been published as a sensitive marker for robotic skill [9]. The other console was connected to the robot and used in a laparoscopic pelvic trainer model (Figure 6). The learners then performed a robotic anastomosis with a Van Velthoven suture (double armed 3-0 Monocryl) using the LapED 4 : 1 model as previously described [10, 11].

## 3. Results

### 3.1. Ease of Model Construction

The cost of the open urological surgery simulators is minimal, and the supplies can be purchased at a local grocery store. The prostatectomy model and wound closure model may be reused for simulation settings, although repeated use may necessitate replacing the orange and banana components for the models, respectively.

### 3.2. Assessing the Models

Ten urology resident trainees attended the session; 7 urology resident trainees completed the questionnaire, ranging from postgraduate year (PGY) two to five. Trainees felt overall that the activities were helpful in practicing their surgical skills (median Likert score 4/5, range 4-5) and that they would recommend these activities to others interested in practicing their surgical skills (median Likert score 4/5, range 4-5). They also felt confident they could easily replicate the activities, excluding the DVSSS, for personal use (median Likert score 4/5, range 4-5). Face validity is typically demonstrated by a Likert score of 4/5 [12]. When assessing the models individually, trainees felt positive about the prostatectomy model's relative realism (median Likert score 4, range 4-5) and its ability to improve their confidence in suturing a prostate (median Likert score 4, range 3-4). They felt ambivalent about the usefulness of the wound closure station in improving their confidence performing the task (median Likert score 3/5, range 3-4). Trainees also felt neutral about the knot-tying station (median Likert score 3.5/5, range 3-4).

## 4. Discussion

Simulators currently available for urology training range from low-fidelity bench models to high-fidelity virtual reality models. The models used during the skills workshop for this study may be considered low fidelity, but have the advantage of being affordable, replicable, and portable for trainee use. Additionally, the simulators in this study focused on improving techniques for open urological procedures, which contrasts the more widely available simulators for laparoscopic and robot-assisted procedures [13].

Options for open technique training are limited; however, there are some other models being developed. An open pyeloplasty model using chicken skin demonstrated effectiveness in improving knot tying and suturing ability among medical students [14]. This model is inexpensive and sufficiently replicates living tissue, but there was significant preparation required in producing the model, though it could easily be reproduced by urology resident trainees.

Low-fidelity models have also been produced for radical prostatectomy using ballistics gel to reproduce neurovascular bundle dissection [15]. The model was significant for its correct anatomy and for emphasizing the anatomical relationships between the fascia, prostatic capsule, and neurovascular bundle. Preparing low-fidelity models may require more preparation but can be performed by attending physicians and their learners at low cost. The same study created a simple prostatectomy model similar to the one utilized in our surgical skills workshop. The model utilized citrus fruit to simulate the prostate and included a balloon pulled through the neck of the fruit to form the prostatic urethra. In addition to the balloon urethra, the model also differed from ours, in that the fruit was sutured to a foam board rather than being fixed within a milk carton to replicate the surgical field. Both the neurovascular bundle model and the simple prostatectomy model demonstrated high Likert scores (4.75/5 and 4.5/5, respectively) for overall learning experience.

Suture simulators may be homemade like those utilized in this workshop, or they may be commercially purchased. Homemade simulators are often made of foam, cloth, fruit, or raw chicken, which either lack realism or are difficult to store long term [16]. Commercial simulators are high fidelity but are often ten-fold more expensive than homemade simulators. The suture simulators used in this workshop included a banana (to replicate wound closure) and cooked pasta (to replicate delicate tissue). Cooked pasta has been used in training plastic surgery trainees for microsurgical techniques and has been demonstrated to be a cost-effective, ethical, and suitable simulator [8]. To the best of the authors' knowledge, the use of cooked pasta for urological training purposes has not been published and may be a viable option for practicing anastomoses of the fragile tissue.

Overall, the trainees considered the various models used in the workshop to be realistic and the activities useful for training purposes. Some activities such as the knot-tying station and wound closure model might be better utilized in the earlier years of surgical training. Only one respondent was a PGY-2 trainee; so, the lower Likert score for these activities may be because the participants had adequate training and self-confidence prior to the workshop.

Despite evaluating face validity, this study did not assess construct validity. Before using the models and exercises for training, it would be advisable to have them assessed by urology experts to evaluate construct validity. This study also did not investigate if training with the simulator translates to improved trainee performance in the operating room. In future studies, we will test improvement on the robot simulator and improved suturing skills in surgery based on performance evaluations.

## 5. Conclusions

This study provides support for exploring innovative methods for surgical skills simulation as it establishes face validity while minimizing costs. The models demonstrated reasonable realism, and the residents endorsed use of the models and activities for improving surgical skills. While these simulators may not fully replace the use of other costlier training options, the authors hope urology residents will take advantage of these affordable alternatives to supplement their training.

## Figures and Tables

**Table 1:** Distribution of responses to face validity questionnaire.

	Strongly disagree	Disagree	Neutral	Agree	Strongly agree	Median Likert score
(1) The orange-in-milk-jug prostate model provided a reasonable representation of an open prostatectomy.				3	2	4
(2) The orange-in-milk-jug prostate model improved my confidence in suturing the prostate during an open prostatectomy.			2	3		4
(3) The banana peel suturing station provided a reasonable representation of tissue behavior.			2	3		4
(4) The banana peel suturing station improved my confidence in suturing for wound closure.			3	1		3
(5) The cooked pasta provided a reasonable representation of delicate tissue behavior.			1	3		4
(6) Suturing the cooked pasta improved my confidence in suturing delicate tissue.			1	3		4
(7) Tying knots with cooked spaghetti helped me determine the appropriate amount of force to apply when advancing a knot.			1	1		3.5
(8) Performing the thread the rings exercise on the Da Vinci skills simulator improved my confidence in controlling a needle laparoscopically.			1	3		4
(9) Overall, I found the activities to be helpful in practicing my surgical skills.				3	2	4
(10) I feel confident that I could easily replicate the activities for personal use (excluding the Da Vinci skills simulator).				3	2	4
(11) I would recommend these activities to others interested in practicing their surgical skills.				3	2	4

## Data Availability

Data are available on request.
